# Assembly of novel microbial genomes from gut metagenomes of rhesus macaque (*Macaca mulatta*)

**DOI:** 10.1080/19490976.2023.2188848

**Published:** 2023-03-15

**Authors:** Shengzhi Yang, Zhenxin Fan, Jiawei Li, Xinqi Wang, Yue Lan, Bisong Yue, Miao He, Anyun Zhang, Jing Li

**Affiliations:** a Key Laboratory of Bioresources and Eco-Environment (Ministry of Education), College of Life Sciences, Sichuan University, Chengdu, Sichuan, China; b Sichuan Key Laboratory of Conservation Biology on Endangered Wildlife, College of Life Sciences, Sichuan University, Chengdu, Sichuan, China; c Institute of Blood Transfusion, Chinese Academy of Medical Sciences, Chengdu, Sichuan, China; d Animal Disease Prevention and Food Safety Key Laboratory of Sichuan Province, Key Laboratory of Bio-Resource and Eco-Environment of Ministry of Education, College of Life Sciences, Sichuan University, Chengdu, Sichuan, China

**Keywords:** *Macaca mulatta*, Metagenomic binning, MAG, non-human primate, gut microbiome, *Campylobacter*

## Abstract

Rhesus macaque (RM, *Macaca mulatta*), as an important model animal, commonly suffers from chronic diarrheal disease, challenging the breeding of RMs. Gut microbiomes play key roles in maintaining intestinal health of RMs. However, it is still unclear about more features of gut microbiome as responsible for intestinal health of RMs. In this study, we performed de novo assembly of metagenome-assembled genomes (MAGs) based on fecal metagenomes from chronic diarrheal RMs and asymptomatic individuals. In total of 731 non-redundant MAGs with at least 80% completeness were reconstructed in this study. More than 97% MAGs were novel genomes compared with more than 250,000 reference genomes. MAGs of *Campylobacter* and Helicobacteraceae from RM guts mainly carried flagella-associated virulence genes and chemotaxis-associated virulence genes, which might mediate motility and adhesion of bacteria. Comparing to MAGs of *Campylobacter* from humans, distributions and functions of these MAGs of *Campylobacter* from RMs exhibited significant differences. Most members of Bacteroidota, Spirochaetota, Helicobacteraceae, Lactobacillaceae and *Anaerovibrio* significantly decreased in guts of chronic diarrhea RMs. More than 92% MAGs in this study were not contained in 2,985 MAGs previously reported from other 22 non-human primates (NHPs), expanding the microbial diversity in guts of NHPs. The distributions and functions of gut microbiome were prominently influenced by host phylogeny of NHPs. Our results could help to more clearly understand about the diversity and function of RMs gut microbiome.

## Introduction

Due to many populations and the similarity of genetic evolution to human, rhesus macaque (RM, *Macaca mulatta*) generally serves as an important model animal to reveal physiology of human, research clinical disease and develop new drugs.^1–3^ Therefore, RMs with huge needs are widely raised in captivity as model animal. However, many captive RMs usually suffer from chronic diarrhea with high morbidity and mortality to pose serious challenges in breeding and management of RMs.^4^ Chronic diarrhea of RMs is prominently manifested by long-term and recurrent diarrhea.^5^ Previous studies indicated that chronic diarrhea disease of RMs was accompanied by significant changes of gut microbial composition and metabolic function.^6,7^ The significant decrease of carbohydrate-active enzyme (CAZyme) genes and significant increase of antibiotic resistance genes (ARGs) were found in guts of chronic diarrhea RMs comparing with asymptomatic RMs.^7^ However, these changes of microbial abundance and diversity might not mean that emergence and continuation of diarrhea disease are caused by the increased/decreased microbiome. In addition, taxonomic annotation of gut microbiome based on metagenomic short reads might lose many important microbial taxa due to databases or algorithms.^6^ The abundance changes of different genes are also difficultly located to concrete microbial species. It is still unclear about more features of gut microbiome as responsible for chronic diarrhea of RMs. Therefore, functional exploration of gut microbiome at genome level is necessary to better understand the important roles of gut microbiome in RMs.

Although some isolates were obtained from guts of RMs in several previous studies,^7,8^ the number of these isolates was negligible in guts of RMs with innumerable microorganisms. For now, bacterial genomes were mainly obtained by conventional culture in laboratory. However, the culture of gut microorganisms in artificial culture medium is still difficult, although many microorganisms have been indiscriminately cultured and were obtained draft genomes.^9–12^ Metagenomic binning is an effective method for reconstruction of genomes from rare community members. The metagenome-assembled genomes (MAGs), the microbial draft genomes assembled from metagenomic reads, have been largely reconstructed from gastrointestinal tracts of animals, such as cow rumen,^13^ chicken cecum,^14^ murine gut.^15^ These MAGs from gastrointestinal tracts of animals vastly promoted the understanding about diversity and function of microbiome. In addition, metagenomic assembly could obtain a mass of novel draft genomes.^16^ These novel MAGs have expanded the life tree to further enlarge the understanding of unknown microorganisms’ characteristics and functions, in particular non-culturable species.^17^

Manara et al.^18^ reconstructed 2,985 MAGs from 22 non-human primate (NHP) species. These MAGs immensely expanded the understanding of diversity and function for NHP gut microbiome, but didn’t involve in MAGs from RMs. The gut microbial composition and diversity, in particular specific microbial taxa, could be partly influenced by host phylogeny.^19,20^ The host specificity of NHPs could reportedly shape the different gut microbial composition.^21–23^ Heritable difference of gut microbiome at genome level might stochastically evolve in adapting of diverse gut environment.^24,25^ These differences of gut microbiome might be presented not only by gut microbial composition but also by genome variation. Therefore, these gut microbial genomes from NHPs could enhance the understanding of host-microbiome coevolution.

In this study, we sought to reconstruct MAGs of RMs (including asymptomatic RMs and chronic diarrhea RMs) based on fecal metagenomic reads to explore the diversity and function of gut microbiome at genome level. We finally obtained 731 non-redundant MAGs from fecal metagenomes of RMs, including many novel strains and novel species comparing with reference genomes, which further expanded genomes sets of NHPs’ gut microbiome. Therefore, our results might help to more clearly understand about the gut microbiome of RMs, in particular chronic diarrhea individuals, more importantly, and provide new insights in prevention for diarrhea of RMs.

## Results

### Reconstruction of 731 MAGs from fecal metagenomes of RMs

Metagenomic binning of 29 fecal metagenomes of RMs (including 11 chronic diarrhea RMs and 18 asymptomatic RMs) from our previous study^7^ were performed. After preliminarily completed metagenomic binning, we obtained 7,082 raw bins from fecal metagenomes of RMs. After removing redundant bins with ANI>99% between genomes, we further obtained 731 non-redundant MAGs with estimated completeness>80% and estimated contamination<10%, 93 MAGs of which were from co-assembly binning and 638 MAGs from single-sample binning. Of these MAGs, 354 MAGs had completeness>90% and contamination<5% (defined as high-quality draft genomes^26^), 145 MAGs had completeness>95% and contamination<5%, and 24 MAGs had completeness>97% and none contamination (Supplementary Figure S1a and Supplementary Table S1).

**Figure 1.**
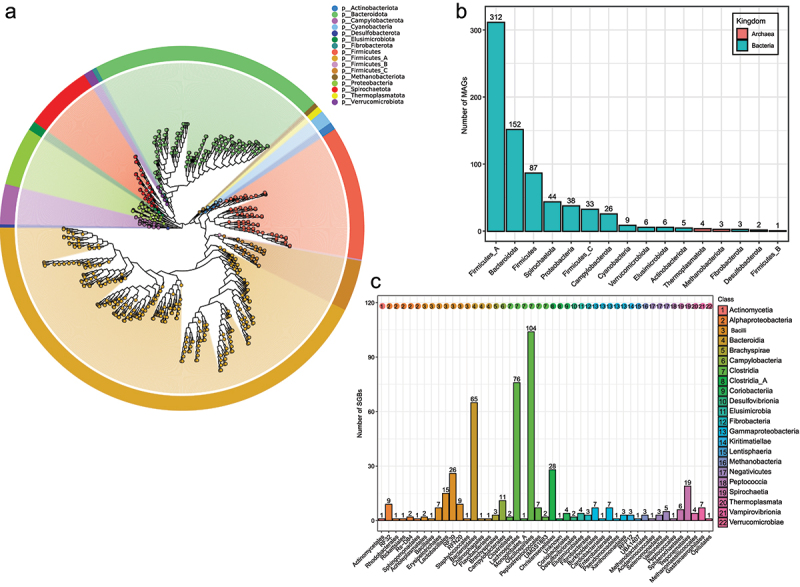
The taxonomic labels of MAGs from fecal metagenomes of RMs. (a) the phylogenetic tree of 731 MAGs from fecal metagenomes of RMs. (b) the number of 731 MAGs at different phyla. (c) the number of SGBs at order level.

Taxonomic labels of all MAGs were identified to class level at least. All 731 MAGs were identified to 2 kingdoms, 16 phyla and 22 classes, 730 MAGs to 46 orders, 728 MAGs to 80 families, 698 MAGs to 219 genera and 464 MAGs to 250 species (Table 1 and Supplementary Table S2). The phylogenetic tree of the 731 MAGs were constructed based on more than 400 most conserved proteins from microbial genomes (Figure 1a). The taxonomic labels of these MAGs at phylum level were consistent with the phylogenetic tree. Of these MAGs, 724 MAGs and 7 MAGs were classified to 14 bacterial phyla and 2 archaeal phyla, respectively. Most MAGs belonged to Firmicutes_A (*n* = 312), followed by Bacteroidota (NCBI taxonomy: Bacteroidetes, *n* = 152), Firmicutes (*n* = 87), Spirochaetota (NCBI taxonomy: Spirochetes, *n* = 44), Proteobacteria (*n* = 38), Firmicutes_C (*n* = 33) and Campylobacterota (NCBI taxonomy: Epsilonproteobacteria, *n* = 26) (Figure 1b). MAGs of Spirochaetota (23/26) and Campylobacterota (40/44) were mainly from asymptomatic RMs. Seven MAGs of archaea consisted of 4 MAGs of Thermoplasmatota and 3 MAGs of Methanobacteriota.

**Table 1.** The taxonomic classification of MAGs in different levels.

Taxonomic level	Classified MAGs	Identified taxa	Unclassified MAGs
Kingdom	731	2	0
Phylum	731	16	0
Class	731	22	0
Order	730	46	1
Family	728	80	3
Genus	698	219	33
Species	464	250	267

The members of Firmicutes_A belonged to the class Clostridia (*n* = 276) and Clostridia_A (*n* = 36) (Supplementary Figure S1b). The members of Clostridia included the order Oscillospirales (*n* = 143), Lachnospirales (*n* = 115), Peptostreptococcales (*n* = 13), Clostridiales (*n* = 2), UMGS1883 (*n* = 2) and Monoglobales_A (*n* = 1). The members of Clostridia_A included the order Christensenellales (*n* = 35) and one undefined order. All members of Bacteroidota belonged to the class Bacteroidia, including the order Bacteroidales (*n* = 150), Flavobacteriales (*n* = 1) and Chitinophagales (*n* = 1). All members of Firmicutes belonged to the class Bacilli, including the order RF39 (*n* = 28), Erysipelotrichales (*n* = 24), Lactobacillales (*n* = 21), RFN20 (*n* = 10), Acholeplasmatales (*n* = 2), Bacillales (*n* = 1) and Staphylococcales (*n* = 1).

Comparing with reference genomes of GTDB, 712 MAGs (97.4%) had ANI<99%. Supplementary Table S3 showed average identity and proportion of assigned proteins of MAGs aligned to UniProt TrEMBL database by MAGpy. Of these MAGs not classified to species level, 250 MAGs (34.2%) had average identity of amino acid<95% and proportion of assigned proteins<90%, defined as potential novel species. These potential novel species belonged to 19 classes of 15 phyla, mainly included 93 MAGs of class Clostridia, 30 MAGs of class Bacilli, 29 MAGs of class Bacteroidia, 24 MAGs of class Spirochaetia, 19 MAGs of class Campylobacteria and 11 MAGs of class Clostridia_A (Supplementary Figure S1b). Of these MAGs not classified to genus level, 32 MAGs had average identity of amino acid<90% and proportion of assigned proteins<90% assigned by MAGpy, mainly including 12 MAGs of Clostridia, 8 MAGs of Campylobacteria, 4 MAGs of Bacilli and 3 MAGs of Clostridia_A.

All bins were also clustered at threshold of 95% ANI to obtain 464 species-level genomes bins (SGBs). Therefore, these SGBs represented taxa of the 731 MAGs at species level. These SGBs mainly belonged to the order Oscillospirales (*n* = 104), Lachnospirales (*n* = 76), Bacteroidales (*n* = 65), Christensenellales (*n* = 28) and RF39 (*n* = 26) (Figure 1c). 103 SGBs had more than one MAG, whilst 361 SGBs only had one MAG. The SGB named as SGB_116 (classified as *UBA636 sp900546285*) had most MAGs (*n* = 17), followed by SGB_73 (*RC9 sp900546445*, *n* = 15), SGB_391 (*Anaerovibrio sp900548165*, *n* = 12) and SGB_29 (*Prevotella sp900548195*, *n* = 10).

### The functional characterization of 731 MAGs

To further compare the functional features, we predicted and annotated the genes of these MAGs. Contigs’ numbers in these 731 MAGs ranged from 3 to 375 (Supplementary Figure S2a and Supplementary Table S1). Total length of MAGs’ contigs ranged from 745,063bp to 4,276,366bp with average length of contigs from 5219.6bp to 498,8330bp. Numbers of predicted genes in MAGs ranged from 720 to 4,080, 88.1% of which ranged from 1,300bp to 2,700bp. rRNA genes of most MAGs cannot be identified, while 5S rRNA genes of 393 MAGs, 16S rRNA genes of 115 MAGs and 23S rRNA genes of 60 MAGs were identified from genome sequences. 47 MAGs included full-length 16S rRNA genes, while 68 MAGs only carried fragment of 16S rRNA genes.

After completed prediction of genes, 1,384,640 genes were contained in 731 MAGs in total. We put together all genes of 731 MAGs to construct non-redundant gene sets at 99%, 95% and 90% identity, producing 1,028,834, 892,970, 835,294 clusters, respectively. The non-redundant gene set at 95% identity were further annotated at protein levels. 841,916 (94.3%) genes were mapped to UniProt TrEMBL database with average identity of 77.0%, whilst 51,054 (5.7%) genes cannot be annotated to UniProt TrEMBL database. In addition, 823,700 (92.2%) genes, 820,072 (91.8%) genes, 764,549 (85.6%) genes, 609 (0.068%) genes and 29,648 (3.3%) genes could be assigned to UniRef90, UniRef50, eggNOG, CARD and CAZy database, respectively.

**Figure 2.**
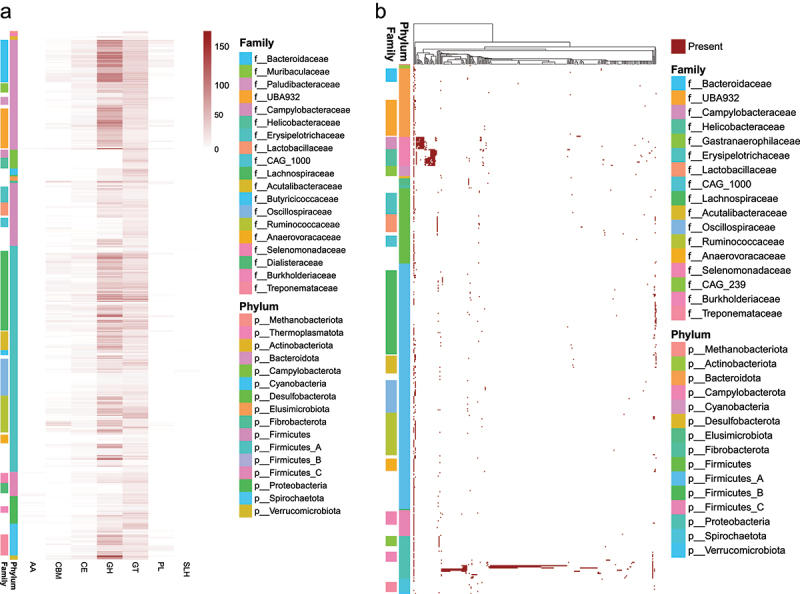
The distribution of CAZymes and virulence genes in MAGs. (a) the distribution of CAZymes genes in MAGs; Glycosyltransferases (GTs); Carbohydrate-binding modules (CBMs); Carbohydrate esterases (CEs); Polysaccharide lyases (PLs); Auxiliary activities (AAs); S-layer homologies (SLHs). (b) the distribution of virulence genes in MAGs.

We predicted CAZymes genes in genome of each MAG. Total 46,057 CAZymes genes were identified from all MAGs. Verrucomicrobiota, Fibrobacterota, Bacteroidota and Firmicutes_A carried most abundant CAZymes genes, and had high percentage of CAZymes genes in genomes (Supplementary Figure S2b). Glycoside hydrolases (GHs) were enriched in the family Bacteroidaceae, UBA932, Lachnospiraceae, Acutalibacteraceae, Ruminococcaceae and UBA1067 (Figure 2a). Glycosyltransferases (GTs) were prevalent in all MAGs. Carbohydrate-binding modules (CBMs) mainly harbored in the family Bacteroidaceae, Lachnospiraceae and Ruminococcaceae. Carbohydrate esterases (CEs) mainly harbored in the phylum Bacteroidota and Firmicutes_A. Polysaccharide lyases (PLs) mainly harbored in the phylum Bacteroidota. Auxiliary activities (AAs) and S-layer homologies (SLHs) were scarce in most MAGs.

We predicted ARGs in genome of each MAG based on CARD database. Total 1,004 ARGs were obtained from 574 MAGs with average identity of 62.2%. Trimethoprim-resistant dihydrofolate reductase dfr was most abundant ARG family (*n* = 474), followed by resistance-nodulation-cell division (RND) antibiotic efflux pump (*n* = 125), major facilitator superfamily (MFS) antibiotic efflux pump (*n* = 67), APH(6) (*n* = 43) and ABC-F ATP-binding cassette ribosomal protection protein (*n* = 28) (Supplementary Table S4). Notably, MAGs of *Escherichia* carried most ARGs. Two MAGs of *Escherichia flexneri* carried 53 (1.3%) and 49 ARGs (1.3%), respectively, and one MAG of *Escherichia albertii* carried 39 ARGs (1.1%).

The virulence-associated genes in genome of each MAG were predicted based on VFDB database. Total 289 virulence genes were identified in MAGs. Notably, the MAGs of Campylobacteraceae, Helicobacteraceae, *Escherichia*, *Haemophilus* and *Haemophilus_A* enriched many virulence-associated genes (Figure 2b).

### The genomes of *Campylobacter* and Helicobacteraceae

Previous studies reported that *Campylobacter* infection, in particular *C. coli* and *C. jejuni*, was associated with diarrhea of RMs.^8,27,28^ In this study, we reconstructed 11 MAGs of *Campylobacter* not assigned to any species level (classified to 2 SGBs, 4 from SGB_80 and 7 from SGB_81). Four MAGs (all belonging to SGB_80) were significantly more abundant in guts of asymptomatic RMs than those in guts of chronic diarrhea RMs (*p* < 0.05), while other 7 MAGs (all belonging to SGB_81) were more abundant in guts of chronic diarrhea RMs, 3 MAGs of which were significantly more abundant (*p* < 0.05) (Supplementary Table S5).

Manara et al.^18^ also reconstructed 3 MAGs of *Campylobacter* from other NHPs. To identify genetic relationship of the 14 MAGs of *Campylobacter*, we downloaded 37 reference genomes of *Campylobacter* from NCBI, and constructed a phylogenetic tree of these genomes (Supplementary Figure S3a). The 11 MAGs of RM and one MAGs of *Macaca fascicularis* were clustered to 2 clades corresponding with taxonomic labels of SGBs. And they were more closely related to *C. hyointestinalis*, *C. fetus* and *C. iguaniorum*, but were distant to *C. coli* and *C. jejuni*.

**Figure 3.**
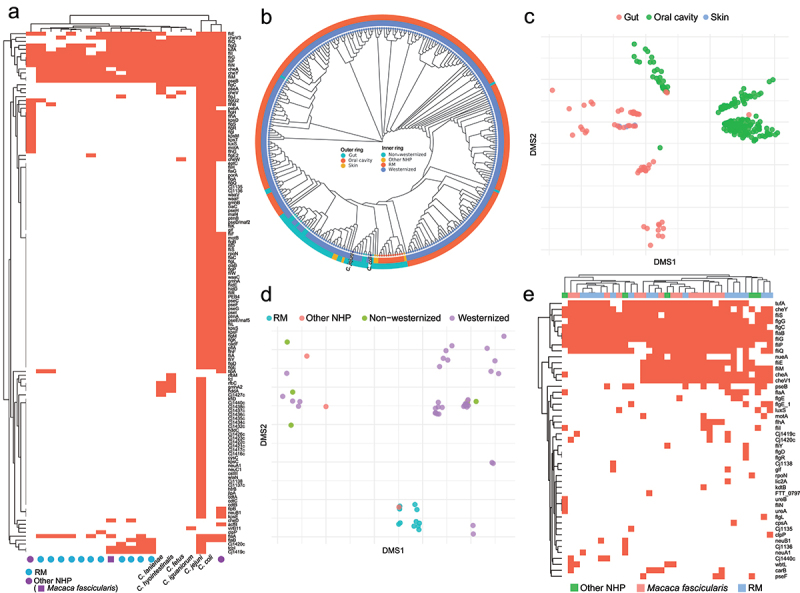
The genomes of *Campylobacter* and Helicobacteraceae. (a) the annotation of virulence genes in genomes of *Campylobacter* from RMs, other NHPs and 6 reference genomes. (b) the phylogenetic tree of MAGs of *Campylobacter* from RMs, other NHPs and humans with different diets. (c) Ordination on functional annotations of MAGs of *Campylobacter* from RMs, other NHPs and humans based on different sites of sampling. (d) Ordination on functional annotations of MAGs of *Campylobacter* from feces based on different hosts including RMs, other NHPs and humans with different diets. (e) the annotation of virulence genes in genomes of Helicobacteraceae from RMs and other NHPs.

We annotated virulence genes in 14 MAGs of *Campylobacter* from RMs and other NHPs, and reference genomes of *C. coli*, *C. jejuni*, *C. fetus*, *C. hyointestinalis*, *C. iguaniorum* and *C. lanienae*. A total of 133 virulence genes were identified in these genomes (Figure 3a). *C. jejuni*, *C. coli* and one MAG of other NHPs harbored most virulence genes, but majority of virulence genes were not detected in other MAGs and other reference genomes. Most MAGs carried *flaA* or *flaB* gene, as the major flagellin genes. The virulence genes of RM’s MAGs were enriched in flagella-associated genes, such as *fliI*, *fliG*, *fliP*, *fliN* and *fliM*. In addition, chemotaxis-associated genes, such as *cheV3*, *cheA* and *cheY*, were also detected in these MAGs.

To further compare differences of *Campylobacter* genomes between NHPs and humans, 301 *Campylobacter* MAGs from humans were obtained from previous study.^29^ These human MAGs were mainly from oral cavities and guts of humans with westernized diet, whilst only 9 MAGs were from oral cavities and guts of humans with non-westernized diet. We constructed a phylogenetic tree of these *Campylobacter* genomes (Figure 3b). All MAGs from RMs and one MAGs from *Macaca fascicularis* were clustered in an independent clade, and 2 other NHP MAGs were close to them. They were significantly different from MAGs of humans in phylogenetic tree. We also functionally annotated genes of these MAGs based on UniRef50 database. NDMS plot exhibited that these MAGs at functional level were obviously separated based on sampling sites, mainly oral cavity and gut (Figure 3c). We further compared functional differences of all MAGs from guts. NDMS plot exhibited that MAGs from RMs at functional level were significantly separated with MAGs from humans (Figure 3d).

*Helicobacter* may be an important factor in development of chronic diarrhea of RMs.^28,30^ However, *Helicobacter* was commonly also prevalent in guts of asymptomatic RMs and significantly decreased in guts of diarrheal RMs.^6,27^ In this study, we retrieved 15 MAGs of Helicobacteraceae, 6 of which belonged to *Helicobacter_B*, 1 to *Helicobacter_C* and 8 not assigned to genus level. All MAGs were recovered from fecal metagenomes of asymptomatic RMs. These MAG of Helicobacteraceae were scarcely detected in fecal metagenomes of chronic diarrhea RMs (only 1 MAG was detected), but were prevalent in fecal metagenomes of asymptomatic RMs.

In addition, 20 MAGs of Helicobacteraceae were also retrieved from metagenomes of other NHPs,^18^ including 12 of which belonged to *Helicobacter_B*, 2 to *Helicobacter_C* and 6 not assigned to genus level. Of these 20 MAGs, 15 MAGs of Helicobacteraceae were recovered from fecal metagenomes of *Macaca fascicularis*. We downloaded 44 reference genomes of *Helicobacter* from NCBI, and constructed a phylogenetic tree of them and MAGs of Helicobacteraceae from RMs and other NHPs (Supplementary Figure S3b). These MAGs were mainly placed in 2 clusters. All 18 MAGs assigned to *Helicobacter_B* were most closely related to *Helicobacter macacae*. 6 MAGs from RMs and one MAG from *Macaca fascicularis* were clustered with reference genome of *Helicobacter macacae*. Notably, 16 of these 18 MAGs were retrieved from RM and *Macaca fascicularis*. Most of MAGs not assigned to genus level were closely related to *Helicobacter winghamensis* and *Helicobacter pullorum*. Only five MAGs of Helicobacteraceae were obtained from metagenomes of humans,^29^ as could suggest that the species of Helicobacteraceae seemed to be rare in human guts comparing with NHPs’ guts.

We also annotated the virulence genes in all MAGs of Helicobacteraceae (Figure 3e). *flaA* or *flaB* genes were detected in most MAGs. Similarly, virulence genes of these MAGs were enriched in flagella-associated genes, such as *fliS*, *flgG*, *flgC*, *flaB*, *fliG*, *fliP* and *fliQ*, and chemotaxis-associated genes, such as *cheY*, *cheA* and *cheV1*.

### Abundances of MAGs in fecal metagenomes of RMs

**Figure 4.**
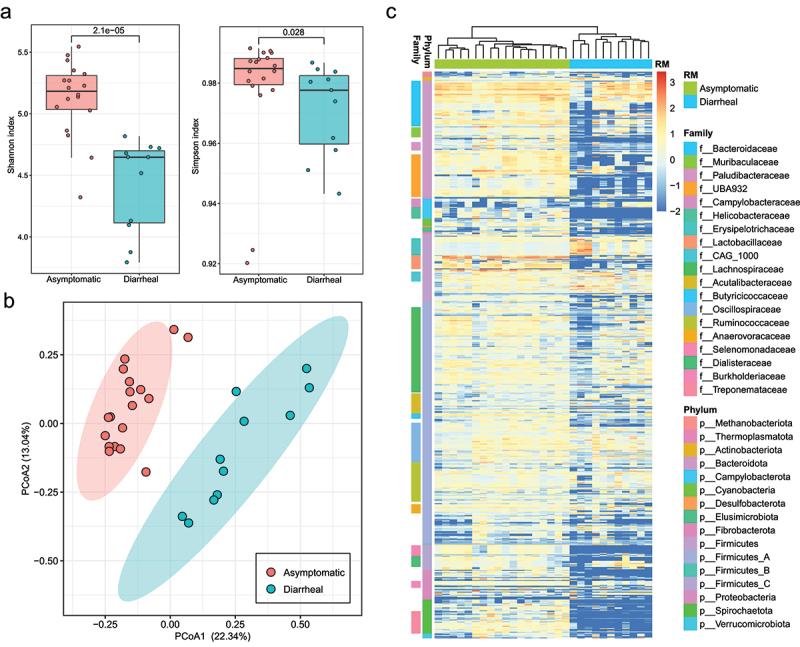
The abundance comparison of MAGs in fecal metagenomes of chronic diarrhea RMs and asymptomatic RMs. (a) the alpha-diversity (Simpson index and Shannon index) of 731 MAGs in guts of asymptomatic and chronic diarrhea RMs. (b) PCoA plot based on Bray-Curtis distance of abundance of 731 MAGs in guts of asymptomatic RMs and chronic diarrhea RMs. (c) heatmap of the abundance of 731 MAGs in fecal metagenomes of asymptomatic RMs and chronic diarrhea RMs.

To compare compositional differences of gut microbiome between asymptomatic RMs and chronic diarrhea RMs, we quantified and compared abundances of the 731 MAGs in fecal metagenomes of RMs. The α-diversity of these MAGs including Shannon index and Simpson indexes were significantly higher in guts of asymptomatic RMs than chronic diarrhea RMs (Figure 4a). Based on Bray-Curtis distance of abundances of MAGs in fecal metagenomes of RMs, PCoA plot exhibited that samples of chronic diarrhea RMs and asymptomatic RMs were significantly divided (Figure 4b). The abundances of all MAGs in the fecal metagenomes of RMs exhibited that samples of chronic diarrhea RMs and asymptomatic RMs were clearly divided in heatmap (Figure 4c). The abundances of 425 MAGs in fecal metagenomes of chronic diarrhea RMs and asymptomatic RMs exhibited significant different (Wilcoxon rank sum test, *p* < 0.05) (Supplementary Table S5). Most members of the phylum Bacteroidota (such as these families UBA932, Paludibacteraceae, Tannerellaceae and P3, as well as some members of Bacteroidaceae), most members of the family Helicobacteraceae, most members of the family Lactobacillaceae (such as *Lactobacillus*, *Ligilactobacillus* and *Limosilactobacillus*), most members of the family Selenomonadaceae (*Anaerovibrio*) and most members of the phylum Spirochaetota (such as *Brachyspira* and *Treponema_D*) were significantly more abundant in guts of asymptomatic RMs than those in guts of chronic diarrhea RMs. Most obviously, several taxa of these MAGs, such as Helicobacteraceae, *Anaerovibrio* of Selenomonadaceae and *Brachyspira* of Brachyspiraceae, were hardly detected in fecal metagenomes of chronic diarrhea RMs, but were prevalent in fecal metagenomes of asymptomatic RMs.

### Comparison of MAGs between RMs and other NHPs

**Figure 5.**
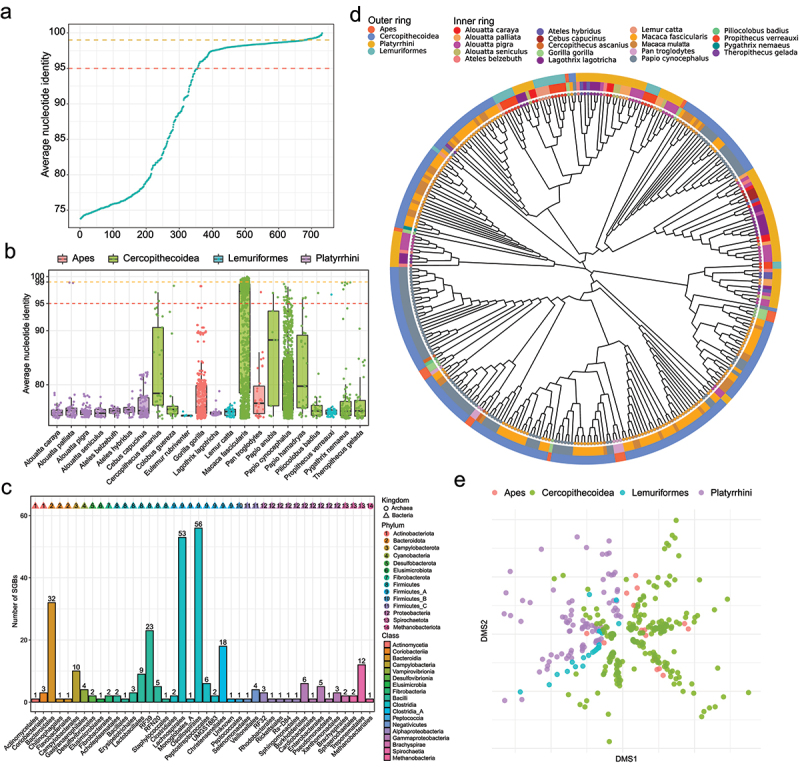
The comparison of MAGs between RMs and other NHPs. (a) the ANI of MAGs of RMs comparing with MAGs of other NHPs. The green dot is the most matching ANI of each MAGs. The red line is 95% ANI threshold, and the orange line is 99% ANI threshold. (b) the ANI distribution of MAGs of other NHPs comparing with MAGs of RMs. (c) the number of MAGs from RMs that were non-redundant compared with MAGs of other NHPs. (d) the phylogenetic tree of *Prevotella* from NHPs. (e) Ordination on functional annotations of MAGs of *Prevotella* from NHPs, based on clades of NHPs.

Manara et al.^18^ had retrieved 2,985 MAGs from 22 NHP species but not including RM. To assess uniqueness of MAGs from RMs comparing with MAGs from other NHPs, ANIs were calculated between the 731 MAGs of RMs and the 2,985 MAGs of other NHPs. 678 MAGs (92.7%) of RMs had ANI<99% as threshold for same strains, whilst 354 MAGs (48.4%) of RMs had ANI<95% as threshold for same species (Figure 5a). Therefore, these 678 MAGs of RMs represented different strains compared with MAGs of other NHPs, and 354 MAGs of RMs belonged to unique species not found in guts of other NHPs. Of these MAGs of other NHPs, MAGs of Cercopithecoidea, in particular *Macaca fascicularis*, had higher ANIs than Lemuriformes and Platyrrhini (Figure 5b). A total of 108 MAGs of other NHPs, all of which were from *Macaca fascicularis*, had ANI>99% comparing with MAGs of RMs. And 608 MAGs of other NHPs, 565 MAGs (92.9%) of which were from *Macaca fascicularis*, had ANI>95% comparing with MAGs of RMs.

To further identify unique species-level genomes in RM guts, ANIs were calculated between SGBs of RMs and other NHPs MAG. About 60% SGBs (*n* = 286) had ANI<95% comparing with other NHP MAGs. Therefore, these 286 SGBs represented unique species in RM guts. These unique species mainly belonged to these orders, including Oscillospirales (*n* = 56), Lachnospirales (*n* = 53), Bacteroidales (*n* = 32), RF39 (*n* = 23), Christensenellales (*n* = 18), Treponematales (*n* = 12) and Campylobacterales (*n* = 10) (Figure 5c).

We re-identified the taxonomic labels of 2,985 MAGs of other NHPs using GTDB-Tk. Of these genera of other NHP MAGs, the genus *Prevotella* had most MAGs (*n* = 301). In addition, 36 MAGs of *Prevotella* were recovered from fecal metagenomes of RMs. To compare the influence by host phylogeny, we constructed a phylogenetic tree of the 337 *Prevotella* MAGs, which were from 19 species of 4 NHPs’ clades (including Platyrrhini, Cercopithecoidea, Lemuriformes and apes) (Figure 5d). These *Prevotella* MAGs mainly were from guts of Platyrrhini and Cercopithecidae. Most MAGs of RMs and *Macaca fascicularis* had shared clades of phylogenetic tree, whilst most MAGs of *Papio cynocephalus* had unshared clades of phylogenetic tree. And MAGs of Platyrrhini also had unshared clades of phylogenetic tree. Based on functional annotation of Uniref50 database, NDMS plot exhibited that these MAGs were separated based on clades of NHPs (Figure 5e). The MAGs from Lemuriformes, Platyrrhini and Cercopithecidae were clearly separated in NDMS plot.

## Discussion

The microbial genomes can provide a better insight to explore the function of gut microbiome in metabolism, nutrition and disease of host. Several studies^6,31–34^ revealed the gut microbial composition of RMs, but it is necessary to develop a deeper understanding of RMs’ gut microbiome at genomes level, especially chronic diarrhea individuals. The reconstruction of microbial genomes based on metagenomic sequences greatly expands the number and diversity of microbial genomes, in particular uncultivable strains.^26^ Although Manara et al.^18^ retrieved 2,985 MAGs from 22 NHPs, none MAG from RMs was obtained due to lack of RMs’ metagenomes. Therefore, in this study, we reconstructed total 731 MAGs from 29 RMs’ fecal metagenomes and further expanded the number and diversity of microbial genomes from NHPs. About 80% MAGs in the study belonged to these phyla Firmicutes_A, Bacteroidota, Firmicutes and Firmicutes_C. These phyla Firmicutes_A, Firmicutes and Firmicutes_C belonged to the phylum Firmicutes in NCBI taxonomy. The taxonomic distribution of these reconstructed MAGs accorded with the abundance of microbial composition in RMs’ gut according to previous studies based on metagenomic sequencing^6,7^ or 16S rRNA gene amplicon sequencing.^31–34^ It also indicated that genomes of high-abundance microbes had a higher recovery ratio based on metagenomes assembly. For low-abundance microbes, co-assembly is an effective means to reconstruct low-abundance MAGs.^35^ The co-assembly improved the recovery ratio of low-abundance MAGs in this study, and increased reconstructed potential of novel genomes.

The GTDB contains about 250,000 bacterial and archaeal genomes and provides a standardized genome-based taxonomy,^36^ which efficiently helps to identify potential novel genomes and taxa. Comparing with reference genomes of GTDB, more than 95% MAGs were non-redundant genomes and more than 1/3 MAGs belonged to potential novel species in our study. Notably, about half of non-redundant genomes belonged to the class Clostridia and Bacteroidia, which played key roles in degrading complex carbohydrates.^37^ For instance, several taxa of Clostridia, such as Lachnospiraceae and Ruminococcaceae, were reportedly capable of degrading complex carbohydrates to produce short-chain fatty acids (SCFAs), maintaining the colonic health of host.^38,39^ The annotation of CAZymes also indicted more abundant GHs in these taxa, which could imply the great potential in converting polysaccharide to SCFAs.^40^ In addition, previous study demonstrated that ARGs were enriched in guts of chronic diarrhea RMs.^7^ The high-abundance ARGs might be closely associated with frequent usage of antibiotics for treatment of diarrhea. In this study, we found that members of *Escherichia* in guts of RMs were main carriers of ARGs. Therefore, monitoring and control of ARGs might need focus on members of *Escherichia*, especially *Escherichia coli*, regarded as an important indicator of pathogens. These MAGs provided new understanding for specific functions of gut microbiome of RMs.

Diarrhea disease could greatly alter the composition and decrease diversity of gut microbiome.^41^ In this study, based on MAGs abundance in guts, we also demonstrated that chronic diarrhea significantly reduced the diversity of gut microbiome. Several taxa of MAGs showed significant difference between chronic diarrhea RMs and asymptomatic RMs. Most taxa were more abundant in guts of asymptomatic RMs than chronic diarrhea RMs. Of these taxa with significant difference, Bacteroidota mainly consisted of the members that are adept at polysaccharide degradation, due to carrying large numbers of CAZymes genes in genomes.^42,43^ In these MAGs of Bacteroidota in gut of asymptomatic RMs might be favorable for degradation of the indigestible polysaccharide. Comparing with chronic diarrhea RMs, these asymptomatic RMs had more lactic acid bacteria, such as *Lactobacillus johnsonii*, *Lactobacillus amylovorus* and *Ligilactobacillus animalis*. Lactic acid bacteria play probiotic roles in gut of host by means of competition of adhesion in epithelial cells with pathogens, producing antimicrobial peptides and SCFAs, and stimulating host defenses.^44,45^ Several species of *Lactobacillus* have been demonstrated probiotic functions in alleviating diarrhea and decreasing incidence of diarrhea.^46,47^ It suggested that gut of RMs was an important resource pool of probiotics. In these taxa with significant difference, Helicobacteraceae, *Brachyspira* and *Anaerovibrio* were prevalent in guts of asymptomatic RMs, but were hardly detected in guts of chronic diarrhea RMs. The *Anaerovibrio* was considered as the important species assisting in lipid hydrolysis to fatty acids.^48^ Several species of *Brachyspira* were prevalent in NHPs and normally didn’t induce inflammatory response.^49,50^ These bacteria might be prevalent in guts of asymptomatic RMs, but the decrease of these taxa could be caused by antibiotic usage of chronic diarrhea RMs.

The species of *Campylobacter*, in particular *C. jejuni* and *C. coli*, were commonly considered as critical pathogens in leading intestinal disease of human.^51,52^ Although *C. coli* and *C. jejuni* were also reportedly associated with diarrhea of RMs,^8,27,28^ all MAGs of *Campylobacter* from RMs were obviously distant to *C. coli* and *C. jejuni* in phylogenetic tree. MAGs of RMs were also different from MAGs of human. Most MAGs of *Campylobacter* from RMs carried *flaA* and *flaB* genes encoding the major flagellin FlaA and FlaB as important structures of flagella, which plays an important role in motility of bacteria.^53^ In addition, the chemotaxis-associated genes, such as *cheA*, *cheD*, *cheV* and *cheY*, mediated the motile sense and directional move.^53^ The flagella of *Campylobacter* contributed the motility but also invasion.^54^ *fliQ*, *fliG* and *fliP* encoded type 3 secretion system (T3SS), medicating secretion of virulence proteins.^55^ However, the key genes, such as *ciaB* and *ciaC*, encoding *Campylobacter* invasion antigens (Cia) was absent in MAGs of RMs. Comparing with reference genomes of *C. coli* and *C. jejuni*, adhesion-associated genes, such as *cadF* and *capA*, invasion associated genes, such as *ciaB* and *ciaC*, and toxin-associated genes, such as *cdtA*, *cdtB* and *cdtC*, were absent in MAGs of RMs. It might suggest the weaker virulence in these MAGs of RMs comparing with *C. coli* and *C. jejuni*. However, due to flagella-associated genes and chemotaxis-associated genes, these *Campylobacter* spp. not belonging to *C. coli* and *C. jejuni* still might cause diarrhea of RMs and should be paid attention to.

*Helicobacter cinaedi* was reportedly common in guts of captive RMs.^56^ However, we didn’t obtain the genomes of *Helicobacter cinaedi* in this study. These MAGs of Helicobacteraceae were also distant to *Helicobacter cinaedi*. MAGs of Helicobacteraceae also carried the *flaA* or *flaB* genes. Similarly, flagella-associated genes and chemotaxis-associated genes were found in MAGs of Helicobacteraceae from RMs and other NHPs. This also suggested that these species of Helicobacteraceae possessed the important characters of motility and adhesion.^57^ We also found most MAGs of Helicobacteraceae, in particular *Helicobacter macacae*, were found in guts of RMs and *Macaca fascicularis*. Therefore, *Helicobacter macacae* might be more prevalent in guts of *Macaca* monkey. The species of Helicobacteraceae were only found in guts of asymptomatic RMs and absent in guts of chronic diarrhea RMs. Previous study also found that *Helicobacter macacae* was lost in guts of chronic diarrhea RMs.^27^ In general, *Campylobacter* and *Helicobacter* were typically colonized in gut mucosa. The pathological gut of chronic diarrhea RMs might be unfavorable for adhesion of them. Alternatively, antibiotic treatment of chronic diarrhea RMs might reduce abundances of *Campylobacter* and *Helicobacter*. Unfavorable adhesion and antibiotic usage might be main causes that *Campylobacter* and *Helicobacter* were decreased in guts of chronic diarrhea RMs.

Comparing with 2,985 MAGs of other NHPs,^18^ more than 90% MAGs were non-redundant genomes and about half genomes represented different species. These unique genomes suggested that RMs’ gut harbored many unique microbes comparing with guts of other NHPs. Of these unique species, Lachnospiraceae and Ruminococcaceae play key role in degrading carbohydrate to produce short-chain fatty acids (SCFAs).^38,39^ Interestingly, most MAGs belonging to same strains or same species were found in MAGs of *Macaca fascicularis*. It could imply that host phylogeny plays an important role in shaping gut microbial composition.^22^ We found that *Prevotella*, belonging to Bacteroidetes, were dominant taxa in gut of NHPs. *Prevotella* was associated with plant-rich diets of host.^58^ The prevalence of *Prevotella* in guts of most NHPs might be associated with high-fiber diets of NHPs. We found that distribution and function of *Prevotella* were strong correlated with host phylogeny. The differences of *Prevotella* distribution and function were significant exhibited between higher clades instead of species of NHPs. It might suggest that adaptive evolution of *Prevotella* in guts of NHPs might earlier appear than existing phylogenetic evolution of NHPs.

In summary, we performed de novo assembly of 731 MAGs including many novel genomes, covering the shortage of gut microbial genomes from RMs. We found that members of *Campylobacter* and Helicobacteraceae might play potential roles in causing intestinal diarrhea of RMs due to flagella-associated virulence genes mediating motility and adhesion of bacteria. Unique gut microbial genomes in RMs suggested obvious difference in gut microbiome between RMs and other NHPs, and also further expanded the diversity of gut microbial genomes in NHPs. We revealed the distributions and functions of gut microbes in NHPs were prominently influenced by host phylogeny of NHPs. Taken together, our data strengthened the functional understanding of RM gut microbiome at genome level, in particular chronic diarrhea individuals, more importantly, and provide new insights in prevention for diarrhea of RMs. The data also strengthened the functional understanding of NHP gut microbiome at genome level and indicated coevolution between host and gut microbiome in NHPs.

## Materials and methods

### Reconstruction of metagenome-assembled genomes

Metagenomic binning of 29 fecal metagenomes of RMs from our previous study^7^ were performed. The study was approved by the Ethics Committee of College of Life Sciences, Sichuan University (No. 20210308001). The 29 fecal metagenomes of RMs were from 18 asymptomatic RMs and 11 chronic diarrhea RMs (Supplementary Table S6). Raw metagenomic reads were trimmed and filtered to remove adapters, low-quality reads and hosts’ sequences using Trimmomatic^59^ and Bowtie2^60^ as KneadData pipeline (https://github.com/biobakery/kneaddata). The assembly of single sample were performed using MEGAHIT^61^ with the option – min-contig-len 300. To retrieve low-abundance bins as much as possible, co-assembly of diarrheal and asymptomatic samples were separately performed using MEGAHIT with the option – continue –kmin-1pass – k-list 27,37,47,57,67,77,87 –min-contig-len 1000. The co-assembly of asymptomatic samples was performed in two batches with 9 samples per batch. Bowtie2 was used to map metagenomic reads back to these single-sample assemblies and co-assemblies. SAMtools^62^ converted SAM file to BAM file. Metagenomic binning of single-sample assembly and co-assembly was performed using MetaBAT2^63^ with the option – minContigDepth 2 –minContigLength 2000. Raw bins were dereplicated at default threshold of 99% average nucleotide identity (ANI) using dRep^64^ with the option dereplicate_wf -comp 80 -con 10 -str 100 -strW 0. Bins were also dereplicated using dRep at threshold of 95% ANI to obtain SGBs. The ANI between genomes was calculated by FastANI.^65^ The completeness and contamination of all bins were evaluated by CheckM.^66^

### Prediction of taxonomy label of MAGs and quantification of MAGs in metagenomes

The putative taxonomy label of MAGs was predicted using GTDB-Tk^67^ with the ‘classify_wf’ function. GTDB-Tk annotation is based on Genome Taxonomy Database (GTDB) taxonomy.^36^ We also obtained NCBI taxonomy of MAGs using script “gtdb_to_ncbi_majority_vote.py” (available from https://github.com/Ecogenomics/GTDBTk/). The putative taxonomy label of MAGs based on GTDB taxonomy was determined by the lowest taxonomy level of classification result of GTDB-Tk. These MAGs were also analyzed using MAGpy,^68^ which uses a Snakemake pipeline based on freely available bioinformatics softwares, including CheckM, Prodigal,^69^ PfamScan,^70^ Sourmash,^71^ PhyloPhlAn^72^ and DIAMOND BLASTP.^73^ The phylogenetic tree of MAGs was constructed by PhyloPhlAn based on more than 400 most conserved proteins from microbial genomes, and was drawn by Figtree (https://github.com/rambaut/figtree) and GraPhlAn.^74^

The abundance of MAGs in metagenome was quantified using Salmon^75^ with “quant_bins” module of MetaWRAP^76^ to estimate the abundance of each scaffold in each sample, and then compute the average abundance of bin. Metagenomic reads were aligned using BWA MEM^77^ and mapping ratios of reads were calculated using SAMtools.

### The function annotation of MAGs

The genes of MAGs were predicted and translated to amino acid sequence by Prodigal. These non-redundant gene sets at proteins level were constructed using CD-HIT^78^ with similarity of 99%, 95% and 90%. These non-redundant genes were aligned to UniProt TrEMBL, UniRef90 and UniRef50 database using DIAMOND, as well were aligned to eggNOG database^79^ using eggNOG-Mapper.^80^ CAZymes genes were identified using hmmscan^81^ based on dbCAN database.^82^ ARGs were identified using RGI^83^ based on comprehensive antibiotic resistance database (CARD).^83^ KEGG orthologys (KOs) were annotated using KofamKOALA.^84^ The rRNA genes of MAGs were predicted using barrnap (https://github.com/tseemann/barrnap). Virulence-associated genes were annotated using DIAMOND to align to virulence factor database (VFDB)^85^ at thresholds of 70% similarity and e-value<1e-5. MAGs were functionally annotated based on Uniref50 using DIAMOND. The non-metric multidimensional scaling (NMDS) plots were drawn using the metaMDS function based on Jaccard distance.

## Supplementary Material

Supplemental Material

## Data Availability

The data that support the findings of this study are openly available in CNGB Sequence Archive (CNSA) of China National GeneBank DataBase (CNGBdb) with accession number CNP0001810 at https://db.cngb.org/.

## References

[1] Itell HL, Kaur A, Deere JD, Barry PA, Permar SR. Rhesus monkeys for a nonhuman primate model of cytomegalovirus infections. Curr Opin Virol. 2017;25:126–17. doi:10.1016/j.coviro.2017.08.005. 28888133 PMC5659282

[2] Colombo APV, Paster BJ, Grimaldi G, Lourenco TGB, Teva A, Campos-Neto A, McCluskey J, Kleanthous H, Van Dyke TE, Stashenko P. Clinical and microbiological parameters of naturally occurring periodontitis in the non-human primate Macaca mulatta. J Oral Microbiol. 2017;9(1):1403843. doi:10.1080/20002297.2017.1403843. 29805776 PMC5963701

[3] Morissette M, Di Paolo T. Non-human primate models of PD to test novel therapies. J Neural Transm (Vienna). 2018;125(3):291–324. doi:10.1007/s00702-017-1722-y. 28391443

[4] Wang K-Y, Christe KL, Yee J, Roberts JA, Ardeshir A. Rotavirus is associated with decompensated diarrhea among young rhesus macaques (*Macaca mulatta*). Am J Primatol. 2019;81(1):e22948. doi:10.1002/ajp.22948. 30620103 PMC8729819

[5] Westreich ST, Ardeshir A, Alkan Z, Kable ME, Korf I, Lemay DG. Fecal metatranscriptomics of macaques with idiopathic chronic diarrhea reveals altered mucin degradation and fucose utilization. Microbiome. 2019;7(1). doi:10.1186/s40168-019-0664-z. PMC6423747 30885266

[6] Rhoades N, Barr T, Hendrickson S, Prongay K, Haertel A, Gill L, Garzel L, Whiteson K, Slifka M, Messaoudi I. Maturation of the infant rhesus macaque gut microbiome and its role in the development of diarrheal disease. Genome Biol. 2019;20(1):20. doi:10.1186/s13059-019-1789-x. 31451108 PMC6709555

[7] Yang SZ, Liu Y, Yang N, Lan Y, Lan WQ, Feng JY, Yue BS, He M, Zhang L, Zhang AY, et al. The gut microbiome and antibiotic resistome of chronic diarrhea rhesus macaques (*Macaca mulatta*) and its similarity to the human gut microbiome. Microbiome. 2022;10(1). doi:10.1186/s40168-021-01218-3. PMC8827259 35139923

[8] Sestak K, Merritt CK, Borda J, Saylor E, Schwamberger SR, Cogswell F, Didier ES, Didier PJ, Plauche G, Bohm RP, et al. Infectious agent and immune response characteristics of chronic enterocolitis in captive rhesus macaques. Infect Immun. 2003;71(7):4079–4086. doi:10.1128/IAI.71.7.4079-4086.2003. 12819098 PMC162015

[9] Forster SC, Kumar N, Anonye BO, Almeida A, Viciani E, Stares MD, Dunn M, Mkandawire TT, Zhu A, Shao Y, et al. A human gut bacterial genome and culture collection for improved metagenomic analyses. Nat Biotechnol. 2019;37(2):186–192. doi:10.1038/s41587-018-0009-7. 30718869 PMC6785715

[10] Zou YQ, Xue WB, Luo GW, Deng ZQ, Qin PP, Guo RJ, Sun HP, Xia Y, Liang SS, Dai Y, et al. 1,520 reference genomes from cultivated human gut bacteria enable functional microbiome analyses. Nat Biotechnol. 2019;37(2):179–185. doi:10.1038/s41587-018-0008-8. 30718868 PMC6784896

[11] Browne HP, Forster SC, Anonye BO, Kumar N, Neville BA, Stares MD, Goulding D, Lawley TD. Culturing of ‘unculturable’ human microbiota reveals novel taxa and extensive sporulation. Nature. 2016;533(7604):543–546. doi:10.1038/nature17645. 27144353 PMC4890681

[12] Liu C, Zhou N, Du MX, Sun YT, Wang K, Wang YJ, Li DH, Yu HY, Song Y, Bai BB, et al. The mouse gut microbial biobank expands the coverage of cultured bacteria. Nat Commun. 2020;11(1):79. doi:10.1038/s41467-019-13836-5. 31911589 PMC6946648

[13] Stewart RD, Auffret MD, Warr A, Walker AW, Roehe R, Watson M. Compendium of 4,941 rumen metagenome-assembled genomes for rumen microbiome biology and enzyme discovery. Nat Biotechnol. 2019;37(8):953–961. doi:10.1038/s41587-019-0202-3. 31375809 PMC6785717

[14] Glendinning L, Stewart RD, Pallen MJ, Watson KA, Watson M. Assembly of hundreds of novel bacterial genomes from the chicken caecum. Genome Biol. 2020;21(1):21. doi:10.1186/s13059-020-1947-1. 32051016 PMC7014784

[15] Lesker TR, Durairaj AC, Galvez EJC, Lagkouvardos I, Baines JF, Clavel T, Sczyrba A, McHardy AC, Strowig T. An integrated metagenome catalog reveals new insights into the murine gut microbiome. Cell Rep. 2020;30(9):2909–2922. doi:10.1016/j.celrep.2020.02.036. 32130896 PMC7059117

[16] Papudeshi B, Haggerty JM, Doane M, Morris MM, Walsh K, Beattie DT, Pande D, Zaeri P, Silva GGZ, Thompson F, et al. Optimizing and evaluating the reconstruction of Metagenome-assembled microbial genomes. BMC Genomics. 2017;18(1). doi:10.1186/s12864-017-4294-1. PMC5706307 29183281

[17] Parks DH, Rinke C, Chuvochina M, Chaumeil P-A, Woodcroft BJ, Evans PN, Hugenholtz P, Tyson GW. Recovery of nearly 8,000 metagenome-assembled genomes substantially expands the tree of life. Nat Microbiol. 2017;2(11):1533–1542. doi:10.1038/s41564-017-0012-7. 28894102

[18] Manara S, Asnicar F, Beghini F, Bazzani D, Cumbo F, Zolfo M, Nigro E, Karcher N, Manghi P, Metzger MI, et al. Microbial genomes from non-human primate gut metagenomes expand the primate-associated bacterial tree of life with over 1000 novel species. Genome Biol. 2019;20(1):20. doi:10.1186/s13059-019-1923-9. 31883524 PMC6935492

[19] Ley RE, Hamady M, Lozupone C, Turnbaugh PJ, Ramey RR, Bircher JS, Schlegel ML, Tucker TA, Schrenzel MD, Knight R, et al. Evolution of mammals and their gut microbes. Science. 2008;320(5883):1647. doi:10.1126/science.1155725. 18497261 PMC2649005

[20] Youngblut ND, Reischer GH, Walters W, Schuster N, Walzer C, Stalder G, Ley RE, Farnleitner AH. Host diet and evolutionary history explain different aspects of gut microbiome diversity among vertebrate clades. Nat Commun. 2019;10(1):10. doi:10.1038/s41467-019-10191-3. 31097702 PMC6522487

[21] Yildirim S, Yeoman CJ, Sipos M, Torralba M, Wilson BA, Goldberg TL, Stumpf RM, Leigh SR, White BA, Nelson KE, et al. Characterization of the fecal microbiome from non-human wild primates reveals species specific microbial communities. PLoS One. 2010;5(11):e13963. doi:10.1371/journal.pone.0013963. 21103066 PMC2980488

[22] Amato KR, Sanders JG, Song SJ, Nute M, Metcalf JL, Thompson LR, Morton JT, Amir A, McKenzie VJ, Humphrey G, et al. Evolutionary trends in host physiology outweigh dietary niche in structuring primate gut microbiomes. Isme J. 2019;13(3):576–587. doi:10.1038/s41396-018-0175-0. 29995839 PMC6461848

[23] Ochman H, Worobey M, Kuo CH, Ndjango JBN, Peeters M, Hahn BH, Hugenholtz P, Achtman M. Evolutionary relationships of wild hominids recapitulated by gut microbial communities. PLoS Biol. 2010;8(11):e1000546. doi:10.1371/journal.pbio.1000546. 21103409 PMC2982803

[24] Garud NR, Good BH, Hallatschek O, Pollard KS, Gordo I. Evolutionary dynamics of bacteria in the gut microbiome within and across hosts. PLoS Biol. 2019;17(1):e3000102. doi:10.1371/journal.pbio.3000102. 30673701 PMC6361464

[25] Walter J, Ley R. The human gut microbiome: ecology and recent evolutionary changes. Annu Rev Microbiol. 2011;65(1):411–429. doi:10.1146/annurev-micro-090110-102830. 21682646

[26] Bowers RM, Kyrpides NC, Stepanauskas R, Harmon-Smith M, Doud D, Reddy TBK, Schulz F, Jarett J, Rivers AR, Eloe-Fadrosh EA, et al. Minimum information about a single amplified genome (MISAG) and a metagenome-assembled genome (MIMAG) of bacteria and archaea. Nat Biotechnol. 2017;35(8):725–731. doi:10.1038/nbt.3893. 28787424 PMC6436528

[27] Laing ST, Merriam D, Shock BC, Mills S, Spinner A, Reader R, Hartigan-O’connor DJ. Idiopathic colitis in rhesus macaques is associated with dysbiosis, abundant enterochromaffin cells and altered T-cell cytokine expression. Vet Pathol. 2018;55(5):741–752. doi:10.1177/0300985818780449. 29929446

[28] Howell S, White D, Ingram S, Jackson R, Larin J, Morales P, Garcia AP, Hicks C, Hopper K, Wagner J. A bio-behavioral study of chronic idiopathic colitis in the rhesus macaque (*Macaca mulatta*). Appl Anim Behav Sci. 2012;137(3–4):208–220. doi:10.1016/j.applanim.2012.01.003.

[29] Pasolli E, Asnicar F, Manara S, Zolfo M, Karcher N, Armanini F, Beghini F, Manghi P, Tett A, Ghensi P, et al. Extensive unexplored human microbiome diversity revealed by over 150,000 genomes from metagenomes spanning age, geography, and lifestyle. Cell. 2019;176(3):649–662. doi:10.1016/j.cell.2019.01.001. 30661755 PMC6349461

[30] Fox JG, Handt L, Xu S, Shen Z, Dewhirst FE, Paster BJ, Dangler CA, Lodge K, Motzel S, Klein H. Novel *Helicobacter* species isolated from rhesus monkeys with chronic idiopathic colitis. J Med Microbiol. 2001;50(5):421–429. doi:10.1099/0022-1317-50-5-421. 11339249

[31] Chen T, Li Y, Liang J, Li Y, Huang Z. Gut microbiota of provisioned and wild rhesus macaques (*Macaca mulatta*) living in a limestone forest in southwest Guangxi, China. Microbiologyopen. 2020;9(3):e981. doi:10.1002/mbo3.981. 31880067 PMC7066464

[32] Cui YF, Wang FJ, Yu L, Ye HH, Yang GB. Metagenomic comparison of the rectal microbiota between rhesus macaques (*Macaca mulatta*) and cynomolgus macaques (*Macaca fascicularis*). Zool Res. 2019;40(2):89–93. doi:10.24272/j.issn.2095-8137.2018.061. 30127329 PMC6378564

[33] Adriansjach J, Baum ST, Lefkowitz EJ, Der Pol Wj V, Buford TW, Colman RJ, Masternak M. Age-related differences in the gut microbiome of rhesus macaques. J Gerontol A Biol Sci Med Sci. 2020;75(7):1293–1298. doi:10.1093/gerona/glaa048. 32052009 PMC7302168

[34] Zhao JS, Yao YF, Li DY, Xu HM, Wu JY, Wen AX, Xie M, Ni QY, Zhang MW, Peng GN, et al. Characterization of the gut microbiota in six geographical populations of Chinese rhesus macaques (*Macaca mulatta*), implying an adaptation to high-altitude environment. Microb Ecol. 2018;76(2):565–577. doi:10.1007/s00248-018-1146-8. 29372281

[35] Stewart RD, Auffret MD, Warr A, Wiser AH, Press MO, Langford KW, Liachko I, Snelling TJ, Dewhurst RJ, Walker AW, et al. Assembly of 913 microbial genomes from metagenomic sequencing of the cow rumen. Nat Commun. 2018;9(1):870. doi:10.1038/s41467-018-03317-6. 29491419 PMC5830445

[36] Parks DH, Chuvochina M, Chaumeil PA, Rinke C, Mussig AJ, Hugenholtz P. A complete domain-to-species taxonomy for Bacteria and Archaea. Nat Biotechnol. 2020;38(9):1079–1086. doi:10.1038/s41587-020-0501-8. 32341564

[37] Faber F, Baumler AJ. The impact of intestinal inflammation on the nutritional environment of the gut microbiota. Immunol Lett. 2014;162(2):48–53. doi:10.1016/j.imlet.2014.04.014. 24803011 PMC4219934

[38] Vacca M, Celano G, Calabrese FM, Portincasa P, Gobbetti M, De Angelis M. The controversial role of human gut Lachnospiraceae. Microorganisms. 2020;8(4):573. doi:10.3390/microorganisms8040573. 32326636 PMC7232163

[39] Qing Y, Xie H, Su C, Wang Y, Yu Q, Pang Q, Cui F. Gut Microbiome, short-chain fatty acids, and mucosa injury in young adults with human immunodeficiency virus infection. Dig Dis Sci. 2019;64(7):1830–1843. doi:10.1007/s10620-018-5428-2. 30560340

[40] Almeida A, Nayfach S, Boland M, Strozzi F, Beracochea M, Shi ZJ, Pollard KS, Sakharova E, Parks DH, Hugenholtz P, et al. A unified catalog of 204,938 reference genomes from the human gut microbiome. Nat Biotechnol. 2021;39(1):105–114. doi:10.1038/s41587-020-0603-3. 32690973 PMC7801254

[41] Li Y, Xia S, Jiang X, Feng C, Gong S, Ma J, Fang Z, Yin J, Yin Y. Gut microbiota and diarrhea: an updated review. Front Cell Infect Microbiol. 2021;11:625210. doi:10.3389/fcimb.2021.625210. 33937093 PMC8082445

[42] Thomas F, Hehemann JH, Rebuffet E, Czjzek M, Michel G. Environmental and gut bacteroidetes: the food connection. Front Microbiol. 2011;2:93. doi:10.3389/fmicb.2011.00093. 21747801 PMC3129010

[43] Flint HJ, Scott KP, Duncan SH, Louis P, Forano E. Microbial degradation of complex carbohydrates in the gut. Gut Microbes. 2012;3(4):289–306. doi:10.4161/gmic.19897. 22572875 PMC3463488

[44] Do Carmo Ms, Santos CID, Araujo MC, Giron JA, Fernandes ES, Monteiro-Neto V, Do Carmo MS. Probiotics, mechanisms of action, and clinical perspectives for diarrhea management in children. Food Funct. 2018;9(10):5074–5095. doi:10.1039/C8FO00376A. 30183037

[45] Zhang Z, Lv J, Pan L, Zhang Y. Roles and applications of probiotic *Lactobacillus* strains. Appl Microbiol Biotechnol. 2018;102(19):8135–8143. doi:10.1007/s00253-018-9217-9. 30032432

[46] Dong H, Liu B, Li A, Iqbal M, Mehmood K, Jamil T, Chang YF, Zhang H, Wu Q. Microbiome analysis reveals the attenuation effect of lactobacillus from yaks on diarrhea via modulation of gut microbiota. Front Cell Infect Microbiol. 2020;10:610781. doi:10.3389/fcimb.2020.610781. 33665171 PMC7920975

[47] Bian X, Wang TT, Xu M, Evivie SE, Luo GW, Liang HZ, Yu SF, Huo GC. Effect of *Lactobacillus* strains on intestinal microflora and mucosa immunity in *Escherichia coli* O157: h7-induced diarrhea in mice. Curr Microbiol. 2016;73(1):65–70. doi:10.1007/s00284-016-1010-3. 27025726

[48] Prive F, Kaderbhai NN, Girdwood S, Worgan HJ, Pinloche E, Scollan ND, Huws SA, Newbold CJ, Planet PJ. Identification and characterization of three novel lipases belonging to families II and V from *Anaerovibrio lipolyticus* 5ST. PLoS One. 2013;8(8):e69076. doi:10.1371/journal.pone.0069076. 23950883 PMC3741291

[49] Spiller RC, Jalanka J. *Brachyspira* and IBS with diarrhoea: a *Helicobacter pylori* moment? Gut. 2021;70(6):1008–1009. doi:10.1136/gutjnl-2020-323370. 33361347

[50] Smith JL. Colonic spirochetosis in animals and humans. J Food Prot. 2005;68(7):1525–1534. doi:10.4315/0362-028X-68.7.1525. 16013401

[51] Liu F, Ma R, Wang Y, Zhang L. The clinical importance of *Campylobacter* concisus and other human hosted *Campylobacter s*pecies. Front Cell Infect Microbiol. 2018;8:243. doi:10.3389/fcimb.2018.00243. 30087857 PMC6066527

[52] Wieczorek K, Wolkowicz T, Osek J. Antimicrobial resistance and virulence-associated traits of *Campylobacter jejuni* isolated from poultry food chain and humans with diarrhea. Front Microbiol. 2018;9:1508. doi:10.3389/fmicb.2018.01508. 30022977 PMC6039573

[53] Bolton DJ. *Campylobacter* virulence and survival factors. Food Microbiol. 2015;48:99–108. doi:10.1016/j.fm.2014.11.017. 25790997

[54] Guerry P. *Campylobacte*r flagella: not just for motility. Trends Microbiol. 2007;15(10):456–461. doi:10.1016/j.tim.2007.09.006. 17920274

[55] Neal-McKinney JM, Christensen JE, Konkel ME. Amino-terminal residues dictate the export efficiency of the *Campylobacter jejuni* filament proteins via the flagellum. Mol Microbiol. 2010;76(4):918–931. doi:10.1111/j.1365-2958.2010.07144.x. 20398207 PMC2914605

[56] Fernandez KR, Hansen LM, Vandamme P, Beaman BL, Solnick JV. Captive rhesus monkeys (*Macaca mulatta*) are commonly infected with *Helicobacter cinaedi*. J Clin Microbiol. 2002;40(6):1908–1912. doi:10.1128/JCM.40.6.1908-1912.2002. 12037042 PMC130736

[57] Gu H. Role of flagella in the pathogenesis of *Helicobacter pylori*. Curr Microbiol. 2017;74(7):863–869. doi:10.1007/s00284-017-1256-4. 28444418 PMC5447363

[58] Tett A, Pasolli E, Masetti G, Ercolini D, Segata N. *Prevotella* diversity, niches and interactions with the human host. Nat Rev Microbiol. 2021;19(9):585–599. doi:10.1038/s41579-021-00559-y. 34050328 PMC11290707

[59] Bolger AM, Lohse M, Usadel B. Trimmomatic: a flexible trimmer for Illumina sequence data. Bioinformatics. 2014;30(15):2114–2120. doi:10.1093/bioinformatics/btu170. 24695404 PMC4103590

[60] Langmead B, Salzberg SL. Fast gapped-read alignment with Bowtie 2. Nat Methods. 2012;9(4):357–359. doi:10.1038/nmeth.1923. 22388286 PMC3322381

[61] Li D, Liu CM, Luo R, Sadakane K, Lam TW. MEGAHIT: an ultra-fast single-node solution for large and complex metagenomics assembly via succinct de Bruijn graph. Bioinformatics. 2015;31(10):1674–1676. doi:10.1093/bioinformatics/btv033. 25609793

[62] Li H, Handsaker B, Wysoker A, Fennell T, Ruan J, Homer N, Marth G, Abecasis G, Durbin R, Proc GPD. The Sequence Alignment/Map format and SAMtools. Bioinformatics. 2009;25(16):2078–2079. doi:10.1093/bioinformatics/btp352. 19505943 PMC2723002

[63] Kang DD, Li F, Kirton E, Thomas A, Egan R, An H, Wang Z. MetaBAT 2: an adaptive binning algorithm for robust and efficient genome reconstruction from metagenome assemblies. PeerJ. 2019;7:e7359. doi:10.7717/peerj.7359. 31388474 PMC6662567

[64] Olm MR, Brown CT, Brooks B, Jf B. dRep: a tool for fast and accurate genomic comparisons that enables improved genome recovery from metagenomes through de-replication. Isme J. 2017;11(12):2864–2868. doi:10.1038/ismej.2017.126. 28742071 PMC5702732

[65] Jain C, Rodriguez RL, Phillippy AM, Konstantinidis KT, Aluru S. High throughput ANI analysis of 90K prokaryotic genomes reveals clear species boundaries. Nat Commun. 2018;9(1):5114. doi:10.1038/s41467-018-07641-9. 30504855 PMC6269478

[66] Parks DH, Imelfort M, Skennerton CT, Hugenholtz P, Tyson GW. CheckM: assessing the quality of microbial genomes recovered from isolates, single cells, and metagenomes. Genome Res. 2015;25(7):1043–1055. doi:10.1101/gr.186072.114. 25977477 PMC4484387

[67] Chaumeil PA, Mussig AJ, Hugenholtz P, Parks DH, Hancock J. GTDB-Tk: a toolkit to classify genomes with the Genome Taxonomy Database. Bioinformatics. 2020;36:1925–1927. doi:10.1093/bioinformatics/btz848. PMC7703759 31730192

[68] Stewart RD, Auffret MD, Snelling TJ, Roehe R, Watson M, Birol I. Magpy: a reproducible pipeline for the downstream analysis of metagenome-assembled genomes (MAGs). Bioinformatics. 2019;35(12):2150–2152. doi:10.1093/bioinformatics/bty905. 30418481 PMC6581432

[69] Hyatt D, Chen GL, Locascio PF, Land ML, Larimer FW, Hauser LJ. Prodigal: prokaryotic gene recognition and translation initiation site identification. BMC Bioinform. 2010;11(1):119. doi:10.1186/1471-2105-11-119. PMC2848648 20211023

[70] Finn RD, Bateman A, Clements J, Coggill P, Eberhardt RY, Eddy SR, Heger A, Hetherington K, Holm L, Mistry J, et al. Pfam: the protein families database. Nucleic Acids Res. 2014;42(D1):D222–30. doi:10.1093/nar/gkt1223. 24288371 PMC3965110

[71] Brown CT, Irber L. Sourmash: a library for MinHash sketching of DNA. J Open Source Softw. 2016;1(5):27. doi:10.21105/joss.00027.

[72] Segata N, Bornigen D, Morgan XC, Huttenhower C. PhyloPhlAn is a new method for improved phylogenetic and taxonomic placement of microbes. Nat Commun. 2013;4(1). doi:10.1038/ncomms3304. PMC3760377 23942190

[73] Buchfink B, Xie C, Huson DH. Fast and sensitive protein alignment using DIAMOND. Nat Methods. 2015;12(1):59–60. doi:10.1038/nmeth.3176. 25402007

[74] Asnicar F, Weingart G, Tickle TL, Huttenhower C, Segata N. Compact graphical representation of phylogenetic data and metadata with GraPhlAn. PeerJ. 2015;3:e1029. doi:10.7717/peerj.1029. 26157614 PMC4476132

[75] Patro R, Duggal G, Love MI, Irizarry RA, Kingsford C. Salmon provides fast and bias-aware quantification of transcript expression. Nat Methods. 2017;14(4):417–419. doi:10.1038/nmeth.4197. 28263959 PMC5600148

[76] Uritskiy GV, DiRuggiero J, Taylor J. MetaWRAP—a flexible pipeline for genome-resolved metagenomic data analysis. Microbiome. 2018;6(1):158. doi:10.1186/s40168-018-0541-1. 30219103 PMC6138922

[77] Li H, Durbin R. Fast and accurate short read alignment with Burrows–Wheeler transform. Bioinformatics. 2009;25(14):1754–1760. doi:10.1093/bioinformatics/btp324. 19451168 PMC2705234

[78] Fu L, Niu B, Zhu Z, Wu S, Li W. CD-HIT: accelerated for clustering the next-generation sequencing data. Bioinformatics. 2012;28(23):3150–3152. doi:10.1093/bioinformatics/bts565. 23060610 PMC3516142

[79] Huerta-Cepas J, Szklarczyk D, Heller D, Hernandez-Plaza A, Forslund SK, Cook H, Mende DR, Letunic I, Rattei T, Jensen LJ, et al. eggNOG 5.0: a hierarchical, functionally and phylogenetically annotated orthology resource based on 5090 organisms and 2502 viruses. Nucleic Acids Res. 2019;47(D1):D309–14. doi:10.1093/nar/gky1085. 30418610 PMC6324079

[80] Huerta-Cepas J, Forslund K, Coelho LP, Szklarczyk D, Jensen LJ, von Mering C, Bork P. Fast genome-wide functional annotation through orthology assignment by eggNOG-mapper. Mol Biol Evol. 2017;34(8):2115–2122. doi:10.1093/molbev/msx148. 28460117 PMC5850834

[81] Eddy SR, Pearson WR. Accelerated profile HMM searches. PLoS Comput Biol. 2011;7(10):e1002195. doi:10.1371/journal.pcbi.1002195. 22039361 PMC3197634

[82] Zhang H, Yohe T, Huang L, Entwistle S, Wu P, Yang Z, Busk PK, Xu Y, Yin Y. dbCAN2: a meta server for automated carbohydrate-active enzyme annotation. Nucleic Acids Res. 2018;46(W1):W95–101. doi:10.1093/nar/gky418. 29771380 PMC6031026

[83] Alcock BP, Raphenya AR, Lau TTY, Tsang KK, Bouchard M, Edalatmand A, Huynh W, Nguyen ALV, Cheng AA, Liu SH, et al. CARD 2020: antibiotic resistome surveillance with the comprehensive antibiotic resistance database. Nucleic Acids Res. 2020;48:D517–25. doi:10.1093/nar/gkz935. 31665441 PMC7145624

[84] Aramaki T, Blanc-Mathieu R, Endo H, Ohkubo K, Kanehisa M, Goto S, Ogata H, Valencia A. KofamKOALA: kEGG Ortholog assignment based on profile HMM and adaptive score threshold. Bioinformatics. 2020;36(7):2251–2252. doi:10.1093/bioinformatics/btz859. 31742321 PMC7141845

[85] Chen LH, Yang J, Yu J, Ya ZJ, Sun LL, Shen Y, Jin Q. VFDB: a reference database for bacterial virulence factors. Nucleic Acids Res. 2005;33(Database issue):D325–8. doi:10.1093/nar/gki008. 15608208 PMC539962

