# Supplementary material for: Assembly of novel microbial genomes from gut metagenomes of rhesus macaque (Macaca mulatta)
Source: Gut Microbes. 2023 Mar 15;15(1):2188848. doi: 10.1080/19490976.2023.2188848 (PMC10026933; doi:10.1080/19490976.2023.2188848)
Supplement: Supplemental Material [file KGMI_A_2188848_SM0127.zip › Supplemental materials Figure.pdf]

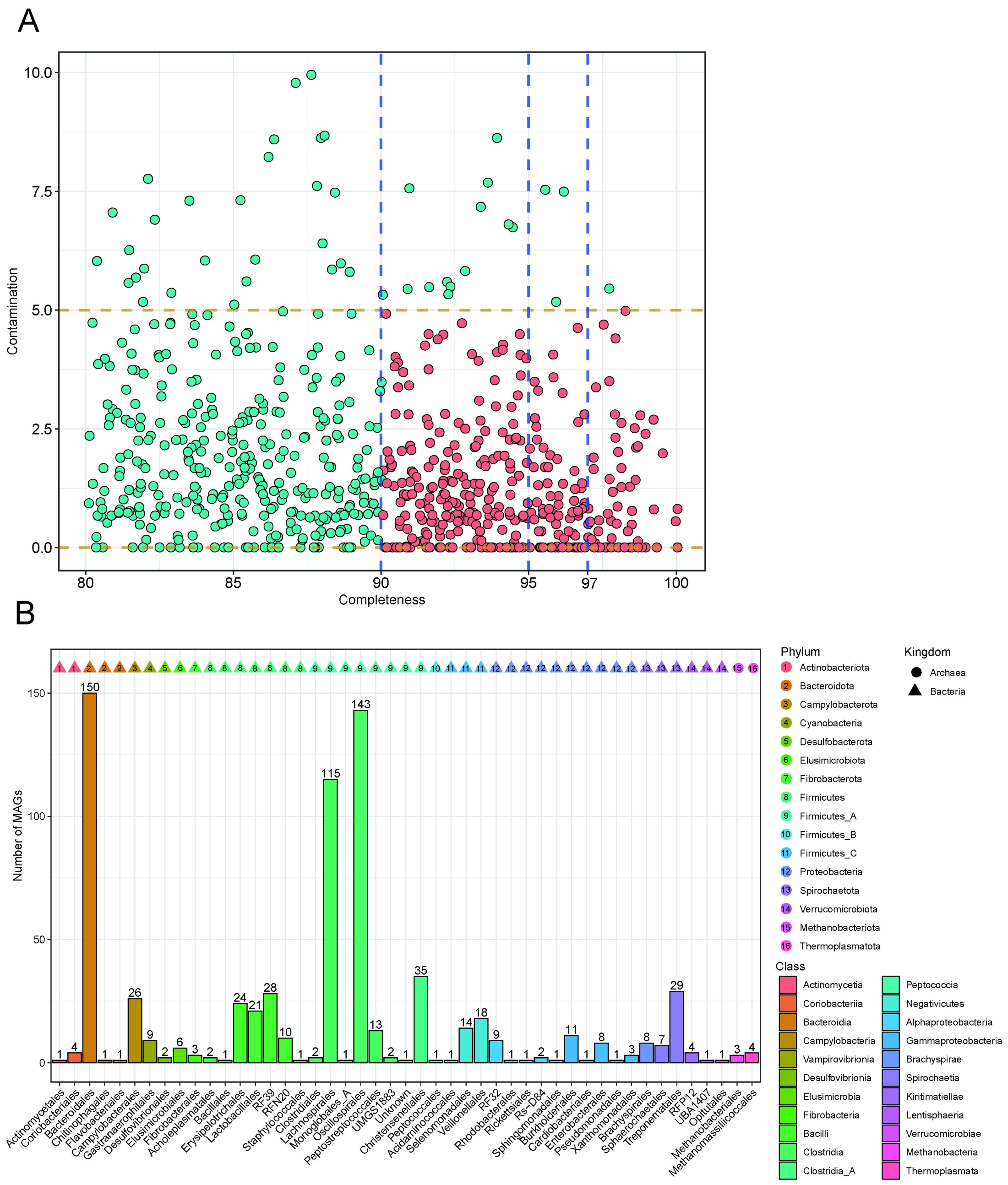

Figure S1 The characterization of MAGs. (a) The distribution of completeness and contamination of MAGs. The red point represents high-quality MAGs with completeness > 90% and contamination < 5%. (b) The number of MAGs at order level.

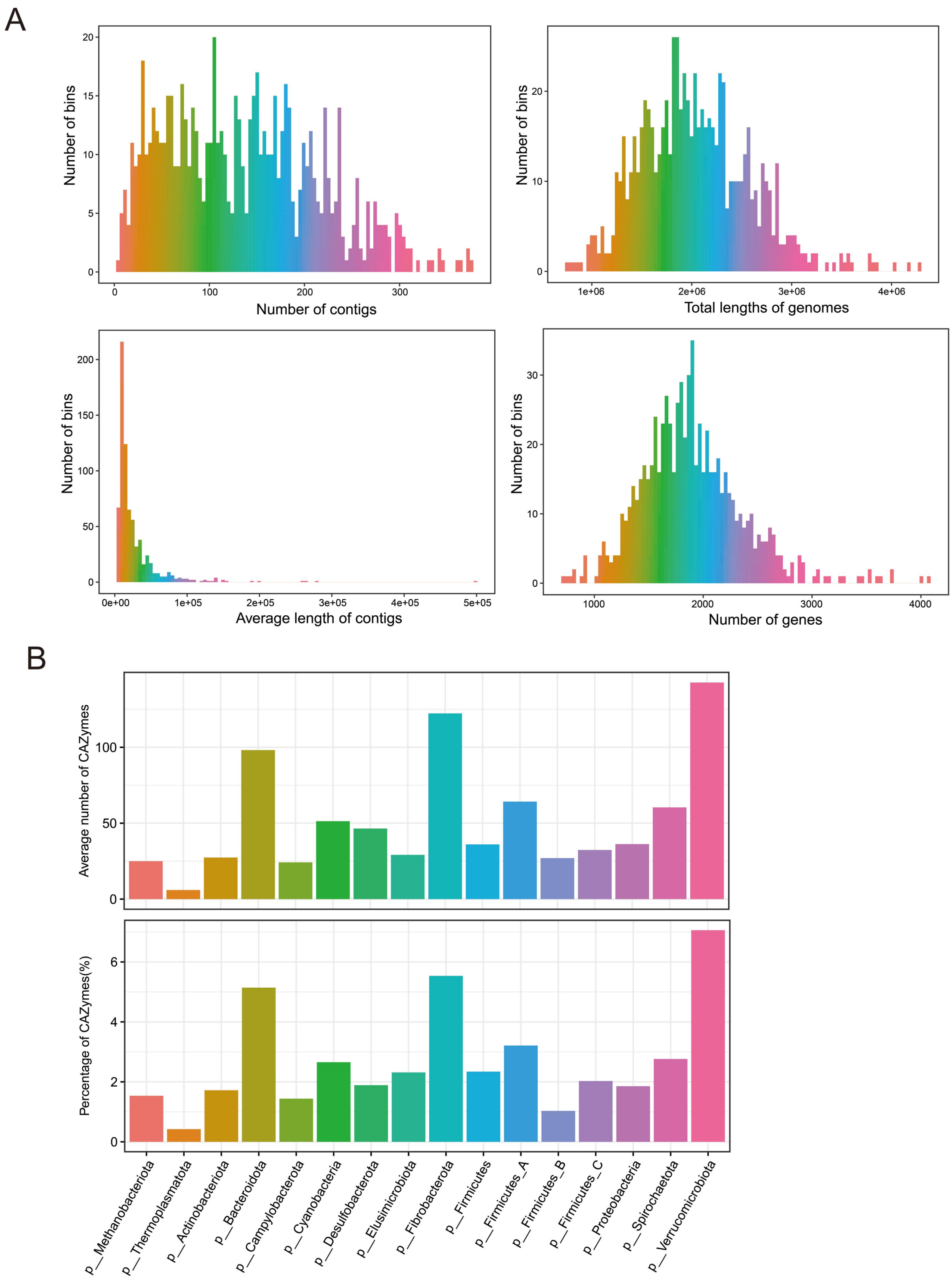

Figure S2 The annotation characterization in MAGs. (a) The distributions of contigs' number (upper left), contigs' total lengths (upper right), contigs' average lengths (low left) and MAGs genes' number (low right). (b) The abundance and percentage of CAZymes in MAGs at different phylum.

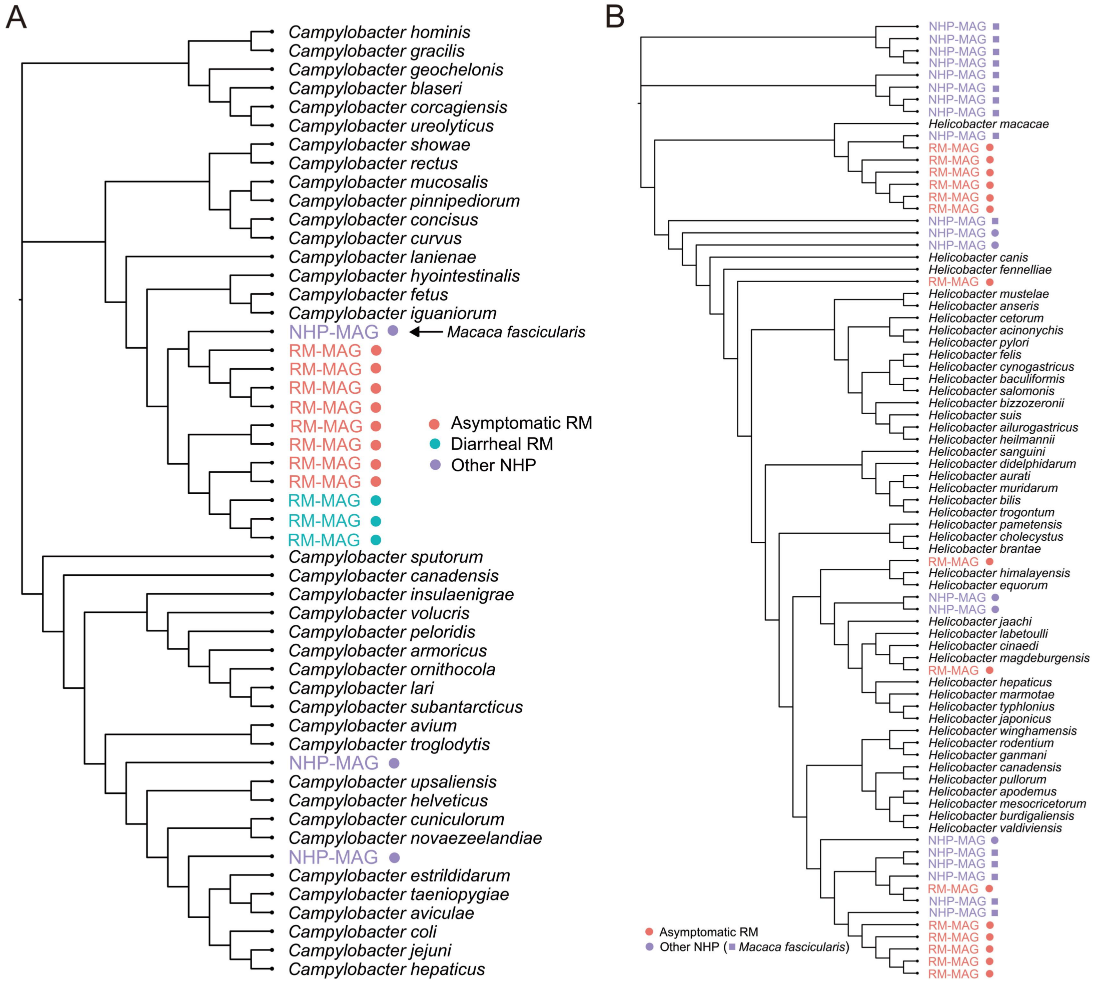

Figure S3 The phylogenetic trees of (a) *Campylobacter* and (b) Helicobacteraceae from RMs and other NHPs.
